# Collateral Impact on Patients of Liver Diseases in the Second COVID-19 Wave: A Retrospective Cohort Study

**DOI:** 10.7759/cureus.25542

**Published:** 2022-05-31

**Authors:** Chitranshu Vashishtha, Ankit Bhardwaj, Amita Diwaker, Shivakshi Sharma, Manoj K Sharma, Shiv Sarin

**Affiliations:** 1 Gastroenterology and Hepatology, Institute of Liver and Biliary Sciences, New Delhi, IND; 2 Epidemiology and Public Health, Institute of Liver and Biliary Sciences, New Delhi, IND; 3 Obstetrics and Gynaecology, Institute of Medical Sciences, Banaras Hindu University, Varanasi, IND; 4 Microbiology, Maulana Azad Medical College, New Delhi, IND; 5 Gastroenterology, Institute of Liver and Biliary Sciences, New Delhi, IND

**Keywords:** morbidity, readmission, pandemic, mortality, liver disease

## Abstract

Background

The second wave of the COVID-19 pandemic in India started in April 2021. This necessitated a change in focus from chronic ailments. This wave lasted till May 2021. Its impact on liver disease patients without COVID-19 infection has not been analyzed.

Methods

Records of liver disease patients from the Institute database admitted from April to May 2021 were compared with that from April to May 2019 i.e., prior to the pandemic. The primary outcome was a comparison of in-hospital mortality rates. Secondary outcomes were a comparison of 30 and 90-day readmission rates and liver transplantation rates.

Results

Seven hundred and seventy-one patients in April-May 2019 (group 1) and 545 patients in April-May 2021 (group 2) were analyzed. Patients in group 2 were sicker with higher PT (INR), urea, creatinine, CTP, and MELD score and low serum sodium, albumin, and platelet count with a higher prevalence of variceal bleed, hepatic encephalopathy, and acute kidney injury. There was higher mortality in group 2 (128/545; 23.5%) than group 1 (124/ 771;16.1%), OR 1.6, 95% CI 1.2 - 2.1, p<0.01. 30 day readmission rate was numerically higher in group1; 18.3% vs 16.9%, p=0.5. The 31-90 day readmission rate was higher in group 1; 29.4% vs 16.9%, p<0.01. There was no significant difference in the number of patients undergoing liver transplantation in two groups, 19 in group 1 and 14 in group 2 (p=0.90).

Conclusion

The second wave of the COVID-19 pandemic had a significant collateral impact on liver disease patients even without causing infection in them. Patients were sicker at the time of admission with higher mortality.

## Introduction

In the current COVID-19 pandemic, several countries have seen multiple peaks of the cases. In India, the first wave was seen in March 2020 with the cases rising gradually. The peak of the first wave of COVID-19 in India appeared on 16th September 2020 with nearly one lac new cases on a day. Cases started to decrease from September 2020 onwards. This was followed by the world’s largest vaccination drive initiated on 16th January 2021 [1]. However, the number of cases began to increase by the end of March 2021, leading to the second wave of the pandemic in April 2021 with exponential increase in the number of cases and subsequent mortality. After reporting more than three-lac cases per day for 10 consecutive days, India reported a whopping more than four-lacs case on a single day on May 1st 2021 [2]. This led Indian health care system to rapidly change the focus from chronic ailments to prevention, diagnosis, treatment and containment of COVID-19 patients. This led to the disruption of the outpatient department (OPD) and inpatient department (IPD) management of various communicable and non-communicable diseases. Private sector health care providers who were not sufficiently equipped to manage COVID-19 cases were shut down. Thus, the existing Indian health care system was overstretched with a potential of increasing the complications and worsening the outcome of patients affected by non-COVID-19 related illnesses [3]. However, the impact of the second COVID-19 wave on patients of liver diseases has not been scientifically analysed. The aim of the study was to assess the impact of second wave of COVID-19 on the disease severity and outcome of patients of liver diseases who were not infected by the COVID-19 virus. We hypothesized that during the COVID-19 second wave, due to reduced health care services availability, patients of liver diseases without COVID-19 infection had worsening of their underlying disease and outcome.

## Materials and methods

Participants and study design

It is a retrospective cohort study conducted at the Institute of Liver & Biliary Sciences, New Delhi, which is a tertiary care hospital for patients with liver diseases in North India. We reviewed hospital records of all consecutive patients of liver without COVID-19 infection from the Institute database who were admitted to the hospital ward from April 2021 to May 2021. As per the institute's policy, patients with negative COVID-19 Rapid Antigen Test and RT-PCR results were admitted to the non-COVID wards and those with positive Rapid Antigen Test or RT-PCR results were admitted into wards dedicated to the care of COVID-19 patients. Also, patients in non-COVID wards who tested positive for COVID-19 infection when they were retested due to suggestive symptoms or signs were shifted to COVID-19 wards and were excluded from the analysis.

Patients with acute liver disease (such as acute hepatitis, acute liver failure - ALF, acute chronic liver failure - ACLF), and chronic liver disease admitted to the hospital were included for study analysis. We used the Asia Pacific Association for the Study of the Liver (APASL) criteria for ACLF in our study. This criteria defines ACLF as an acute hepatic insult presenting as jaundice (serum bilirubin > 5 mg/dl and coagulopathy (INR > 1.5) complicated within four weeks by clinical ascites and/or encephalopathy in a patient with previously diagnosed or undiagnosed chronic liver disease/cirrhosis and is associated with high 28-day mortality. 

Patients with obstructive jaundice, patients with benign biliary and pancreatic diseases, and liver space-occupying lesions as the reason for admission were excluded from the analysis. This data was compared with that from April 2019 to May 2019 i.e., prior to the COVID-19 pandemic. Because of difficulty in access to hospital care, and diversion of the hospital staff and resources, there was a potential of affecting the daycare procedures. Hence the comparison of the daycare procedure data in the two time periods was also analyzed.

The primary outcome of the study was the comparison of in-hospital mortality rates in the two time periods. The secondary outcomes were a comparison of 30-day and 90-day readmission rates after the discharge and liver transplantation rates in the two time periods. The study was approved by our Institutional Ethics Committee (IEC) / Institutional Review Board (IRB) (No. F.37/ (1)/9/ILBS/DOA/2020/20217/568 Dated: 9.12.2021). The study was registered at ClinicalTrials.gov. with identifier number NCT05167305.

Statistical analysis

Baseline parametric data were expressed as the proportion, mean± standard deviation, and median with an interquartile range as appropriate. Categorical variables were analyzed by chi-squared test or Fisher exact test while the continuous variables were analyzed using an unpaired t-test or Mann-Whitney test as appropriate. To assess the factors associated independently with increased mortality, multivariate analysis with backward elimination was used. This is a stepwise regression approach that begins with a full model and then stepwise eliminates variables from the model to ultimately find a reduced model which best explains the outcome. The p-value < 0.05 was considered statistically significant. All statistical tests were performed using IBM Corp. Released 2016. IBM SPSS Statistics for Windows, Version 24.0. Armonk, NY: IBM Corp.

## Results

Overall, 1313 patients were admitted in April-May 2019 (group 1) and 969 patients were admitted in April-May 2021 (group 2). In group 1, 68 patients had malignant obstructive jaundice, 90 had benign biliopancreatic diseases and 25 had benign space-occupying lesions in the liver as the cause of admission and were excluded from the analysis. Similarly, in group 2, 35 patients had malignant obstructive jaundice, 88 had benign biliopancreatic diseases and 45 had benign space-occupying lesions in the liver as the cause of admission and were excluded from the analysis. Hence, 771 patients in group 1 and 545 patients in group 2 were analyzed in the study.

**Figure 1:**
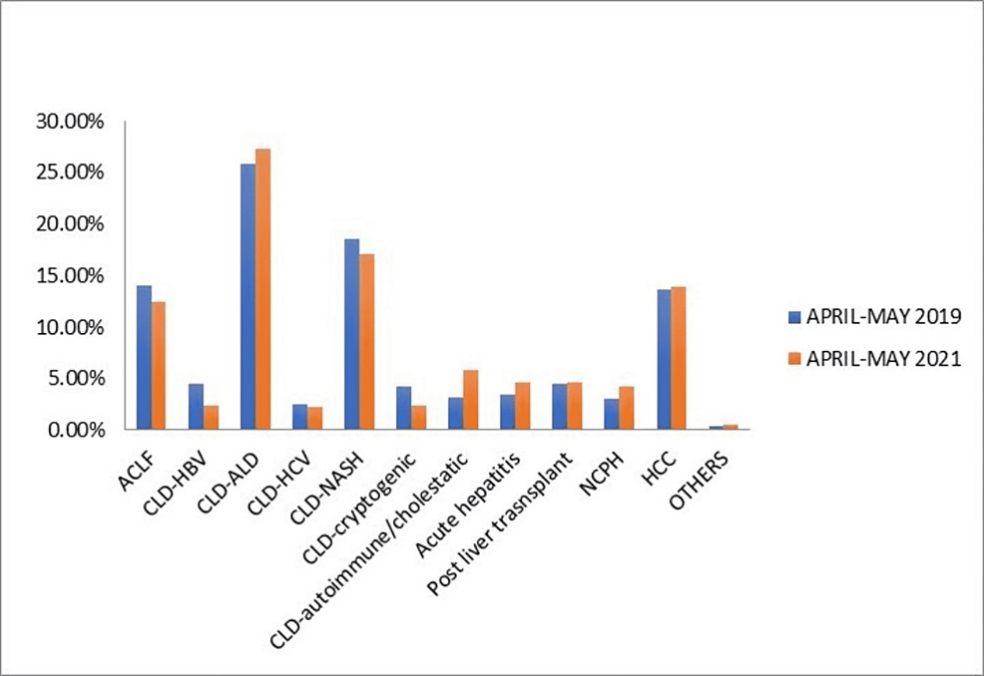
Etiologies of liver disease in the two groups.

There was no significant difference in etiologies of liver disease in the two groups (Figure 1). Most of the patients in both the groups were of chronic liver disease (CLD) with alcoholic liver disease (ALD) being the most common etiology. Among other causes of CLD were hepatitis C virus (HCV), nonalcoholic steatohepatitis (NASH), hepatitis B virus (HBV), and cryptogenic, and autoimmune hepatitis / cholestatic liver disease. The rest of the cases were of ACLF, hepatocellular carcinoma (HCC), non-cirrhotic portal hypertension (NCPH), and post-liver transplantation patients.

Patients in the second group were sicker with higher PT (INR), urea, creatinine, CTP, and MELD score and low serum sodium, serum albumin, and platelet count. These patients also had a higher prevalence of variceal bleed, hepatic encephalopathy, and acute kidney injury at the time of presentation (Table 1).

**Table 1:** Clinical characteristics of the patients admitted in two periods.

Variable	April-May 2019 (n=771)	April-May 2021 (n=545)	p value
Age (years)	50.60±13.70	50.03 ± 14.36	0.47
Gender	M: F = 633:138	M: F = 437:108	0.38
Weight (kg)	72.13 ±16.90	69.12 ± 16.61	<0.01
BMI (kg/m^2^)	26.72 ± 10.26	28.59 ± 65.40	0.54
Etiology ACLF (APASL criteria) CLD-HBV CLD-ALD CLD-HCV CLD-NASH CLD-cryptogenic CLD-autoimmune/cholestatic CLD-Wilson’s disease Acute hepatitis Post Liver transplant Non Cirrhotic Portal Hypertension (NCPH) Hepatocellular carcinoma (HCC) Infiltrative liver disease Liver abscess ALF Mixed etiologies	108 (14%) 35 (4.5%) 200 (25.9%) 19 (2.5%) 143 (18.5%) 32 (4.2%) 25 (3.2%) 2 (0.3%) 27 (3.5%) 35 (4.5%) 24 (3.1%) 105 (13.6%) 3 (0.4%) 1 (0.1%) 4 (0.5%) 8 (1%)	68 (12.5%) 13 (2.4%) 149 (27.3%) 12 (2.2%) 93 (17.1%) 13 (2.4%) 32 (5.9%) 0 (0%) 25 (4.6%) 25 (4.6%) 23 (4.2%) 76 (13.9%) 2 (0.4%) 0 (0%) 4 (0.7%) 10 (1.8%)	0.15
Complications at admission			
Variceal bleed n (%)	46 (6%)	81(15.6%)	<0.01
Acute Kidney Injury n (%)	136 (17.6%)	231 (44.4%)	<0.01
Hepatic encephalopathy n (%)	48 (6.2%)	156 (30%)	<0.01
Sepsis n (%)	236(30.6%)	133(25.6%)	0.05
Laboratory features			
Hemoglobin (gm/dl)	10.21± 2.247	10.06 ± 2.478	0.27
Total Leukocyte Count (TLC) (per cu. ml)	9.18 ± 6.98	9.25 ± 6.56	0.86
Platelet count (per cu. ml)	107 (68.5-162)	71 (49-118)	<0.01
Total bilirubin (mg/dl)	2.90 (1.5-8.8)	3.26 (1.43 – 10.03)	0.25
ALT (IU/l)	36.5 (25.0-62.0)	37.0 (24.0 - 62.0)	0.94
AST (IU/l)	74 (46-129.8)	73.1 (47-128.6)	0.74
s. albumin (gm/dl)	2.97 ± 0.87	2.58 ± 0.74	<0.01
Prothrombin Time (PT) International normalized ratio (INR)	1.65 (1.37 – 2.1)	1.56 (1.27-2.1)	<0.01
s. urea (mg/dl)	34.24(21.4-55.6)	37 (22.9-67.4)	<0.01
s. creatinine (mg/dl)	1.0 (1.0-1.24)	1.0 (1.0-1.53)	0.01
s. sodium (mEq/l)	133.02 ± 6.27	131.90 ± 5.87	<0.01
Child Turcotte Pugh (CTP) score	9.17 ± 2.39	9.47 ± 2.27	0.07
Model for End Stage Liver Disease (MELD) score	18.1 (13.37 – 25.8)	21.0 (14.07 – 33.1)	<0.01

Primary outcome analysis

**Figure 2:**
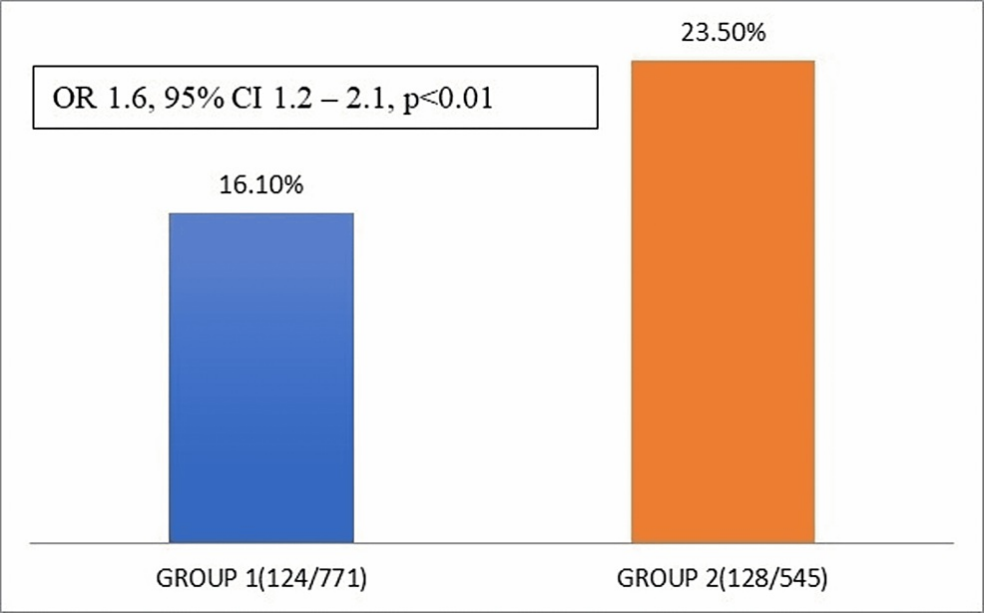
Difference of mortality between two groups.

There was significantly higher mortality in group 2 (128/545; 23.5%) than group 1 (124/ 771;16.1%), odds ratio (OR) 1.6, 95% confidence interval (CI) 1.2 - 2.1, p<0.01 (Figure 2).

In univariate analysis, significant variables (at 5 % significance) which were associated with increased mortality were hemoglobin, total leukocyte count (TLC), total bilirubin, albumin, urea, creatinine, sodium, prothrombin time (PT) international normalised ratio (INR), Child Turcotte Pugh (CTP) score, Model for End-stage Liver Disease (MELD) score, presence of Acute kidney injury (AKI), variceal bleed, Hepatic encephalopathy (HE) and sepsis. These variables were added to the multivariate model and backward elimination was applied. Prognostic composite scores such as CTP and MELD were excluded from the multivariate analysis due to multicollinearity with individual variables. In multivariate analysis, the presence of HE, sepsis, AKI, and high bilirubin were independently associated with increased mortality (Table 2).

**Table 2:** Multivariate analysis, showed the presence of HE, sepsis, AKI and high bilirubin to be independently associated with increased mortality.

	Univariate analysis			Multivariate analysis		
Variable	OR	95% C.I.	p value	OR	95%C.I.	p value
Age (years)	1.00	0.98-1.0	0.06			
Hemoglobin (gm/dl)	0.87	0.82-0.92	0.01			
TLC (per cu. ml)	1.12	1.11-1.14	<0.01			
Total bilirubin (mg/dl)	1.07	1.05-1.08	<0.01	1.03	1.01 to 1.06	0.02
S. Albumin (gm/dl)	0.48	0.40 – 0.59	<0.01			
PT (INR)	2.35	1.98-2.80	<0.01			
S. Urea (mg/dl)	1.02	1.01-1.02	<0.01			
S. Creatinine (mg/dl)	1.7	1.5 – 1.9	<0.01			
Variceal bleed	1.7	1.09 to 2.5	0.02			
AKI	11.1	8.1 – 15.3	<0.01	6.8	3.6 to 12.8	<0.01
HE	7.2	5.2 – 10.0	<0.01	12.0	6.7 to 21.5	<0.01
Sepsis	12.9	9.3 – 17.9	<0.01	10.0	5.473 to 18.4	<0.01
MELD	1.1	1.10 – 1.13	<0.01			
CTP	1.9	1.7-2.1	<0.01			

Secondary outcome analysis

**Figure 3:**
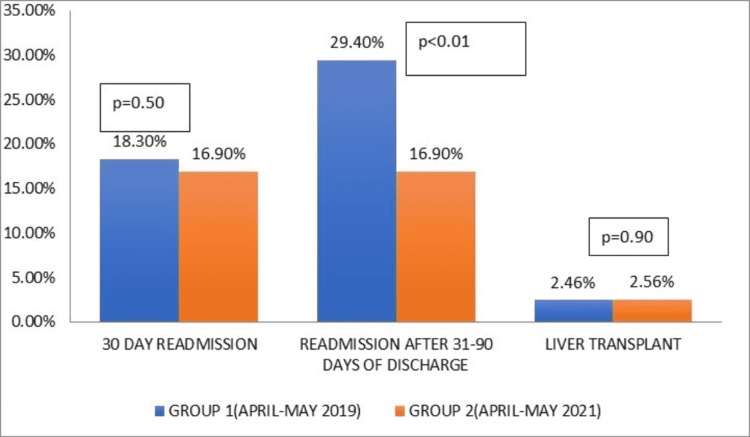
Difference in the 30-day readmission rates in the two groups.

There was no significant difference in the 30-day readmission rates in the two groups and was numerically higher in group 1 [18.3% (141/771) in group 1 and 16.9% in group 2 (92/545), p=0.5]. There was a significantly higher readmission rate of admission in 31-90 days after the discharge in group 1 compared to group 2 [29.4% (227/771) in group 1 and 16.9% (114/545) in group 2], p<0.01. There was no significant difference in the number of patients undergoing liver transplantation in the two groups. 19 patients underwent liver transplantation in group 1 compared to 14 patients in group 2 (p=0.90) (Figure 3).

**Figure 4:**
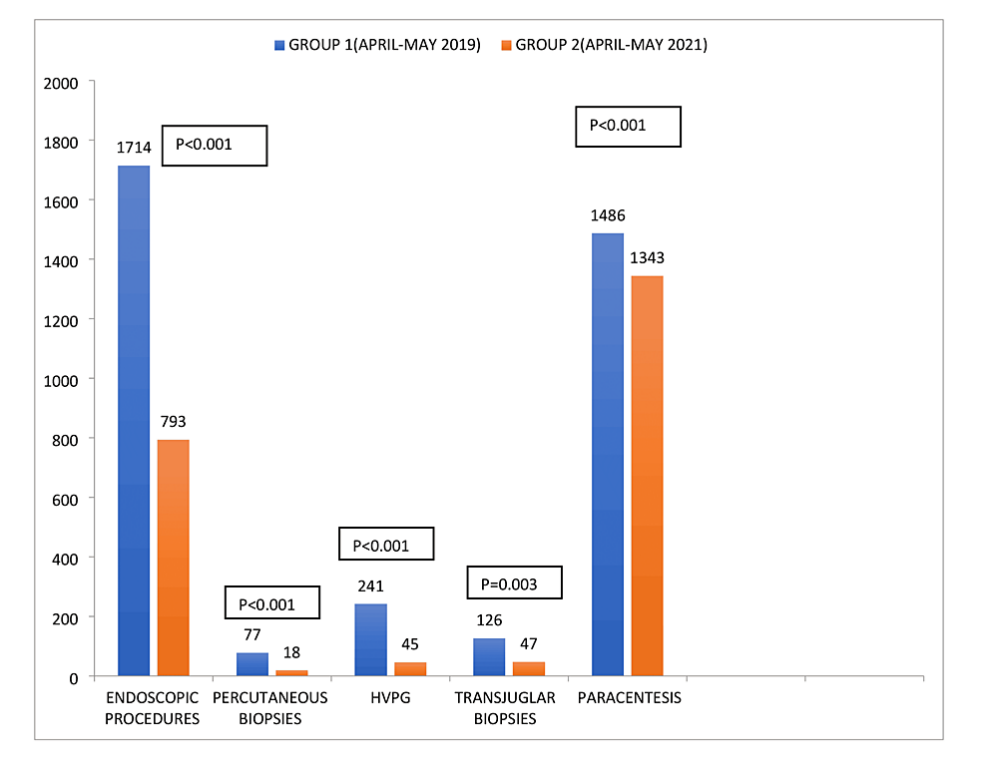
Difference in procedures performed among the two groups.

Analyzing the daycare procedures, in group 1, a total of 1714 endoscopic procedures, 77 percutaneous liver biopsies, 241 hepatic venous pressure gradient (HVPG) measurements, and 126 transjugular liver biopsies were done. In group 2, a total of 793 endoscopic procedures, 18 percutaneous liver biopsies, 45 Hepatic venous pressure gradient (HVPG) measurements, and 47 transjugular liver biopsies were done. During the same time period, the number of large volume paracentesis done was 1486 in group 1 compared to 1343 in group 2 (Figure 4). The overall number of the daycare procedures was significantly higher in group 1 when compared to group 2, p< 0.001. Individually also, the number of procedures in each category of the daycare procedures was significantly more in group 1 when compared to group 2.

## Discussion

Compared to the first wave, even though there was no significant percentage increase in the death rate in the second wave, the total death numbers were disappointingly high due to the very high number of infections [4]. Various studies have identified multiple double mutants and triple mutant strains of the SARS-CoV-2 across different regions of India which were more pathogenic and less susceptible to the vaccines and were one of the important causes of the surge of cases in the second wave [5]. Apart from the mutant strains, other factors such as violation of COVID appropriate behavior, government, and public complacency were the key reasons for the surge in the number of patients [6].COVID-19 vaccination began in India from 16 January 2021 onward in a phased manner. In the first phase, the COVID-19 vaccine was given to the health care and front-line workers. The second vaccination phase started on 1st March 2021, which allowed vaccination of all Indians with age more than 60 years and those with age 45 to 59 years along with comorbidities. From April 1 onward, all people above 45 years of age were eligible for the COVID-19 vaccine [7]. Thus, few patients with liver disease could have the vaccination at the start of the second wave.

Additionally, the sheer number of patients affected by the second wave strained the already fragile Indian health care system. To combat the second wave, isolation beds and wards for COVID-19 suspects and confirmed cases, new temporary hospital setups were built. Hospital staff, equipment, and consumables were diverted to the care of these patients from existing resources. All these measures were effective in countering the second wave but worsened the outcome of patients affected by non-COVID-19-related illnesses. Patients of various other communicable and non-communicable diseases and even routine immunizations were affected. With the cessation of outpatient department services in many healthcare facilities, routine diabetes care was compromised, leading to suboptimal glycaemic control [8]. In liver disease patients, COVID-19 has direct as well as indirect impacts. Directly, COVID-19 infection per se leads to abnormalities in liver metabolism and increased morbidity and mortality in patients with underlying chronic liver disease. In this study, we present, to our knowledge, the first study analyzing the indirect impact of the second wave of COVID -19 pandemic on liver disease patients. Patients in the second wave were having more advanced liver disease at admission with more prevalence of variceal bleed, acute kidney injury, hepatic encephalopathy, and higher MELD score. Consequently, they had higher in-hospital mortality as well. Surprisingly, despite the patients being sicker in the second wave, they had lesser readmission rates. This could be due to difficulty in accessing hospital care in the ongoing pandemic. Similarly, the number of daycare procedures was less in the second COVID-19 wave. This may be due to resource allocation (manpower and equipment) for the care of COVID-19 patients and difficulties in access to hospital care.

Recent reports of new SARS-CoV-2 variants having mutations that enhance multiple key mechanisms which favor host cell entry and viral replication are concerning. This not only increases disease severity and transmission ability of the virus but also reduces neutralization by naturally or vaccine-induced antibodies [9]. Thus, the COVID-19 pandemic is far from over. This study emphasizes the need for proactive measures which should be taken so that the negative consequences, both direct in patients infected by the virus as well as indirect impact on those noninfected but with comorbidities, can be avoided. This should include hospitals being prepared with adequate equipment along with ventilators and oxygen supply and enhanced vaccine coverage, including children. The importance of vaccination and the booster doses should be emphasized to the patients as well as health care professionals. Patients and attendants entering health care facilities should be screened for fever, respiratory or other symptoms suggestive of COVID-19. The use of telemedicine should be encouraged since access to hospital care is difficult for patients suffering from chronic ailments or can expose them to the COVID-19 infection. The current study has several limitations. This is a single-center study. This is a retrospective study, however, prospective studies addressing the issue are not feasible. To compare a relatively homogenous group of patients, only the patients with liver disease who were admitted were analyzed. Thus, the impact on other patients admitted and OPD patients were not analyzed.

## Conclusions

In conclusion, the second wave of COVID-19 pandemic had significant collateral impact on patients of liver disease even without causing infection in them. The patients were sicker at the time of admission and had higher mortality. The study result should be an impetus to take preemptive measures to minimise the negative impact of the ongoing pandemic.
